# Clinical and genetic evidence from East Asian cohorts linking family history burden and menarche timing to curve severity in adolescent idiopathic scoliosis

**DOI:** 10.3389/fendo.2026.1876849

**Published:** 2026-08-07

**Authors:** Ryutaro Tanaka, Takuro Iwami, Yoshiro Yonezawa, Nao Otomo, Kazuki Takeda, Shiro Ikegawa, Morio Matsumoto, Masaya Nakamura, Kota Watanabe, Chikashi Terao

**Affiliations:** 1 Department of Orthopaedic Surgery, Keio University School of Medicine, Tokyo, Japan; 2 Laboratory for Statistical and Translational Genetics, RIKEN Center for Integrative Medical Sciences, Yokohama, Japan; 3 Shizuoka General Hospital, The Clinical Research Center, Shizuoka, Japan; 4 Center for Translational Research, Scottish Rite for Children, Dallas, TX, United States; 5 The Department of Applied Genetics, School of Pharmaceutical Sciences, University of Shizuoka, Shizuoka, Japan; 6 Department of Statistical and Translational Genetics, Center for Genomic Medicine, Fujita Health University, Aichi, Japan

**Keywords:** adolescent idiopathic scoliosis, age at menarche, Cobb angle, family history, genetic correlation, genetic predisposition, genetics, Mendelian randomization

## Abstract

**Introduction:**

Adolescent idiopathic scoliosis (AIS) affects 2% to 3% of adolescents and progresses rapidly during puberty. Previous studies suggest that a positive family history of AIS and a later age at menarche (AAM) are risk factors for curve severity in AIS, but their quantitative evidence and the causal contribution of AAM remain to be elucidated.

**Methods:**

We analyzed 5,891 patients with AIS to evaluate the associations of family history and AAM with curve severity. Genetic correlation, Mendelian randomization, harmonized variant-level analyses, and variance component analyses were also performed.

**Results:**

Family history was associated with curve severity (*β* = 1.26, *P* = 0.024), and this association showed a dose-dependent relationship (*β* = 1.31, *P* = 0.006). Later AAM was linearly associated with greater curve severity (*β* = 1.95, *P* = 3.7 × 10^−23^). AAM showed genetic correlation with Cobb angle (r_g = 0.25, *P* = 0.04), but not with susceptibility to AIS (r_g = 0.04, *P* = 0.33), suggesting distinct contributions of AAM to AIS susceptibility and curve severity. Mendelian randomization provided suggestive evidence for an effect of later AAM on curve severity, with no evidence of horizontal pleiotropy. Harmonized variant-level analyses showed that AIS susceptibility alleles tended to have concordant positive effects on curve severity. A variance component analysis demonstrated that AAM and family history explained 2.7% and 0.2% of the variance in Cobb angle, respectively.

**Discussion:**

This study provides quantitative genetic evidence linking family history of AIS and later AAM to curve severity. These findings support the use of family history and AAM in clinical settings to stratify patients according to the risk of greater curve severity.

## Introduction

Adolescent idiopathic scoliosis (AIS) is a complex three-dimensional spinal deformity defined by a lateral curvature with a Cobb angle (curve severity) ≥10° (1). Its prevalence has been estimated at 2% to 3% among adolescents based on large-scale epidemiological reports (2–4). AIS progression is the most pronounced during periods of rapid growth (5) and carries both physical and psychological burdens (6, 7), including respiratory compromise (8). Management is undertaken with conservative measures or surgical intervention, depending on curve severity. Surgical intervention burden patients and their family; therefore, prediction of progression after developing AIS is strongly desired.

AIS is considered as a multifactorial condition influenced by genetic and environmental factors (9–11). Family aggregation has long been recognized in AIS. First-degree relatives have a higher prevalence of AIS than the general population (12). Our group has identified 20 genome-wide significant loci for AIS (10, 13–16) via genome-wide association studies (GWASs). In addition, a polygenic risk score (PRS) for AIS susceptibility was shown to associate with Cobb angle, suggesting that curve severity carries a polygenic burden shared with AIS susceptibility (17). Since DNA microarray data or sequencing data are not usually available in clinical settings, family history can be an alternative measure to evaluate genetic burdens. While a positive family history of AIS is associated with greater curve severity and a higher likelihood of treatment in a cross-sectional cohort of 1,463 patients with idiopathic scoliosis (18), quantitative analyses between family history of AIS and Cobb angle in a large-scale data set are quite limited.

Furthermore, later age at menarche (AAM) appears to be associated with curve progression in AIS. In a 20-year school-screening-based cross-sectional series of 38,879 patients, later AAM was associated with curve progression (19). Longitudinal cohorts indicate that curve progression is fastest around menarche (20), and a single-center longitudinal cohort of 1,090 untreated small curves shows that curve progression tends to cluster around menarche and that the progression phase lasts longer in girls with later AAM (21). Since AAM reflects estrogen-regulated maturation (22–24) and prior genetic and functional studies implicate estrogen receptor signaling in AIS progression and severity (25–28), the relationship between AAM and curve severity warrants investigation. However, the causal relationship between AAM and AIS curve severity and the quantitative evaluation of the contribution of AAM remain to be elucidated.

Most prior evidence linking family history and AAM to curve severity has come from school screening programs or other cross-sectional studies, which capture Cobb angle at a single time point (18, 19). Capturing the severity of established curves requires data of Cobb angle at last visit (after bone maturation), and comparing associations of the initial and final visit would be informative. However, evidence based on visit after bone maturation and longitudinal assessment has been comparatively limited (20, 21).

To address these gaps, here we analyzed hospital-based clinical data from 5,891 individuals with AIS, quantifying outcomes using the initial Cobb angle and last follow-up Cobb angle and analyzing associations between Cobb angle and family history of AIS or AAM. To evaluate whether the observed clinical links have a genetic basis, we further integrated GWAS summary statistics by performing genetic correlation analyses, Mendelian randomization (MR) analyses for AAM, and a harmonized variant-level analysis of AIS susceptibility and curve severity.

To the best of our knowledge, this is the first study to quantitatively evaluate associations between family history and curve severity in a large AIS data set and genetically investigate the relationship between AAM and Cobb angle.

## Materials and methods

### Ethics approval statement

The study protocol was approved by the Medical Ethics Committee of Keio University Hospital, Tokyo (approval no. 20080129) and the Ethics Committee of RIKEN Center for Integrative Medical Sciences, Yokohama, Japan (approval no. H0243-001).

### Patient consent statement

Written informed consent was obtained from all participants (and/or their legal guardians, where applicable), including consent for the publication of clinical details. All genomic DNAs from patients were examined after obtaining informed consent.

### Subjects and clinical assessment

A total of 5,891 consecutive Japanese patients diagnosed with AIS were included in this study. The patients were recruited from outpatient clinics at participating orthopedic institutions. Both conservative and surgical cases were included. Clinical information and phenotypic measurements were obtained through standardized assessments conducted by board-certified orthopedic surgeons affiliated with the scoliosis research group. Data collected included age, sex, height, weight, body mass index (BMI), AAM, and the therapy that the patient underwent. We measured Cobb angle as a radiological outcome. These variables were assessed at the time of initial visit and at the last follow-up. Regarding the last follow-up, for patients who underwent surgical correction, the values closest to the time of surgery were used. For those managed conservatively, data from the end of follow-up were used. Since Cobb angle at last visit can be regarded as a final outcome, we mainly analyzed Cobb angle at last visit, unless otherwise specified.

### Definition of family history

We conducted a patient survey to assess the family history of AIS up to the fourth degree of kinship (including first cousins) in patients diagnosed with AIS. The survey was administered by orthopedic surgeons through direct interviews with the patients. In cases where the presence or absence of a family history could not be clearly confirmed, patients were categorized as having no family history in order to avoid overestimation. We carefully excluded congenital scoliosis and symptomatic scoliosis from the family history.

### Definition of AIS

One patient was diagnosed as AIS when a structural spinal curve with a coronal Cobb angle of ≥10° was identified on standing radiographs.

### Demographics

The cohort was predominantly female, comprising 5,476 female patients and 372 male patients. Radiographic assessments at the initial visit were available for 3,808 participants, and data at the last follow-up were available for 5,802 participants. Of these, 3,803 had radiographic assessments at both the initial visit and the last follow-up. The mean follow-up duration was 2.8 ± 2.8 years overall, and it was significantly longer in conservatively managed than in surgically treated patients (3.2 ± 2.8 vs. 2.0 ± 2.8 years, *P* < 0.001).

Of the 1,231 patients who had an AIS family history, 1,073 had one close relative with AIS history, 139 had two relatives, and 19 had three relatives (the maximum number of close relatives was three). At the initial visit, the mean age was 13.9 ± 4.5 years overall, 13.9 ± 4.3 years in those without a family history, and 14.1 ± 5.0 years in those with a family history.

We performed analyses using data from 3,808 individuals at the initial visit and 5,802 individuals at the last follow-up. All statistical analyses were performed using R version 4.2.1, The R Foundation for Statistical Computing, Vienna, Austria. Two-sided Welch’s *t*-tests were applied to compare the mean values of each continuous value between the groups with and without family history of AIS (**Table 1**).

**Table 1:** Demographics of the subjects.

Characteristics	Total	FH		*P* value
		(+)	(−)	
Clinical assessment	5880	1231	4649	
Number of close relatives with AIS history				
One	–	1073	–	–
Two	–	139	–	–
Three	–	19	–	–
Sex				
Men	372	77	295	–
Women	5476	1154	4322	–
Age (year): mean ± SD				
in initial visit	13.9±4.5	14.1±5.0	13.9±4.3	0.2
in final follow-up	16.6±4.8	16.6±5.3	16.6±4.7	0.76
BMI (kg/m^2^): mean ± SD				
in initial visit	17.8±2.5	17.7±2.3	17.9±2.5	0.22
in final follow-up	19.2±2.4	19.1±2.3	19.3±2.4	0.05
AAM (year): mean ± SD				
	12.1±1.3	12.2±1.6	12.1±1.2	0.07
Treatment				
Surgery	1803	373	1430	–
Non-surgery	3773	807	2966	–
Cobb angle (^o^): mean ± SD				
at initial visit	35.2±15.1	36.8±16.6	34.7±14.7	0.001*
at final follow-up	38.0±15.8	39±16.1	37.7±15.7	0.01*
Follow-up (year): mean ± SD (n)				
Surgery	2.0 ± 2.8 (1,346)	–	–	< 0.001*
Non-surgery	3.2 ± 2.8 (2,356)	–	–	

Values are shown as mean ± standard deviation unless otherwise indicated. Continuous variables were compared between groups with and without a family history of AIS using two-sided Welch’s t-tests. * P value <.05.

Abbreviations: FH, family history; AIS, adolescent idiopathic scoliosis; BMI, body mass index; AAM, age at menarche.

### Association of family history or the number of affected relatives with Cobb angle in AIS

Assuming that higher Cobb angles reflect greater severity, we compared the Cobb angle at the initial visit and at the last follow-up across groups defined by AIS family history. We first contrasted the presence or absence of family history and then examined categories defined by the number of affected relatives. Because the group with three affected relatives was small, we combined the two and three relative categories and analyzed three ordered groups—zero, one, and two or more affected relatives. To account for potential confounding by age and BMI, Cobb angle at each time point was regressed on age and BMI, and comparisons were performed on the resulting residuals across family history status and across the ordered relative count categories. We additionally fitted multivariable linear regression models including family history status or the ordered relative count as predictors to estimate linear trends in Cobb angle adjusted for age and BMI. In addition, we applied the Jonckheere–Terpstra test to evaluate a monotonic trend across the ordered categories. As a sensitivity analysis, we repeated these analyses separately in conservatively managed and surgically treated patients.

### Association between AAM and Cobb angle in AIS

We examined whether AAM was associated with curve severity. Because age at menarche is defined only in female patients, all AAM-related analyses were restricted to female patients with a recorded AAM (*n* = 4,119). AAM was categorized into three ordered groups: an early group defined as 11 years or younger (*n* = 1,304), a control group defined as 12 to 15 years (*n* = 2,792), and a late group defined as 16 years or older (*n* = 23) (total *n* = 4,119). For each time point, we fitted multivariable linear regression models with the Cobb angle as the dependent variable and the AAM group as the primary predictor, adjusting for age and BMI at the same time point. The AAM group was modeled as an ordinal predictor to capture a linear trend in curve severity across the three AAM categories. To evaluate an overall monotonic trend across the ordered AAM groups, we also used the Jonckheere–Terpstra test. As a sensitivity analysis, we additionally modeled AAM as a continuous variable (years) within the same multivariable framework, adjusting for age and BMI at the same time point. These analyses were also repeated separately in conservatively managed and surgically treated patients.

### Genetic correlation between AAM and curve severity in AIS

Genetic correlations were estimated using bivariate linkage disequilibrium score regression (LDSC) (29, 30). We analyzed summary statistics for AAM, AIS susceptibility, and Cobb angle in AIS. For AAM, we used a large Japanese GWAS of reproductive aging that included up to 67,029 women of Japanese ancestry (31). For AIS susceptibility, we used the Japanese GWAS with 5,327 cases and 73,884 controls (10). For curve severity, we used GWAS summary statistics from an East Asian AIS cohort of 4,465 patients, treating Cobb angle as a curve severity phenotype (21). An East Asian LD reference was used to match the ancestry of the summary statistics. Summary statistics were harmonized following standard LDSC preprocessing, which included restriction to HapMap3 SNPs, alignment to the reference strand, removal of strand ambiguous variants, use of autosomes only, and exclusion of the MHC region on chromosome 6 from 25 to 34 Mb (details are shown in “**Supplementary Materials and Methods**”).

### Genetic analyses

#### Data source

We used GWAS summary statistics for AAM from a multi-ancestry meta-analysis including 799,845 women, of which 166,890 were of East Asian ancestry (32). For curve severity in AIS, we used an East Asian AIS cohort of 4,465 patients (21). For AIS susceptibility, we used a Japanese case–control GWAS totaling 5,327 cases and 73,884 controls (10). This AIS susceptibility GWAS and the curve severity GWAS were also used in a harmonized variant-level analysis assessing the relationship between AIS susceptibility and curve severity. These outcome resources match those used in the genetic correlation analyses and ensure consistent ancestry and preprocessing across methods (details are shown in “**Supplementary Materials and Methods**”).

#### Instrument selection and harmonization

From the AAM meta-analysis, we extracted 1,080 significant signals (*P* < 5 × 10^-8^) as candidate instruments (32). We applied the same three inclusion rules for all outcomes. First, the variants had to be present in an East Asian AAM dataset. Second, the effects had to be concordant between the meta-analysis and the East Asian dataset. Third, the variants also had to be available in the respective outcome summary statistics. Exposure and outcome were harmonized in R using TwoSampleMR (33).

Effect and non-effect alleles were aligned, signs were flipped when required, and ambiguous palindromic variants were removed. We then applied the TwoSampleMR instrument filter and excluded weak instruments using SNP-level *F* statistics defined as *F* = β*x*²/SE(β*x*)² with a threshold of *F* ≥10. This yielded 149 instrument variables for curve severity and 150 for AIS susceptibility. For the variant-level analysis of AIS susceptibility and curve severity, we first selected 20 genome-wide significant variants (*P* < 5 × 10^-8^) from the Japanese AIS susceptibility GWAS as candidate variants. Seventeen of these variants were available in the curve severity GWAS, and their effect alleles were harmonized between the two datasets before downstream analysis (details are shown in “**Supplementary Materials and Methods**”).

#### Primary analysis

Causal effects were estimated with inverse-variance weighting (IVW) as the primary analysis for AAM-based MR analyses (34). Effects are interpreted per unit increase in genetically predicted AAM (years per effect allele). The units are degrees for curve severity and log odds for AIS susceptibility. For the analysis of AIS susceptibility and curve severity, we compared harmonized SNP-specific effect estimates between the two traits using an unweighted linear regression of SNP effects on curve severity against SNP effects on AIS susceptibility (details are shown in “**Supplementary Materials and Methods**”).

#### Sensitivity analyses

Robustness was assessed using MR-Egger regression with an unconstrained intercept and the weighted median estimator (35–37). We conducted single-SNP and leave-one-out analyses. MR-PRESSO was used for the global outlier test, and when outliers were detected we reported an outlier-corrected IVW estimate (38). For the AIS susceptibility–curve severity analysis, we additionally evaluated the sign concordance of harmonized SNP effects between the two traits (details are shown in “**Supplementary Materials and Methods**”).

#### Variance partition analysis of the last follow-up Cobb angle

We estimated the proportion of variance in the last follow-up Cobb angle explained by family history, AAM, BMI, and age using variance partitioning in R (variancePartition). We fitted a fixed-effects linear model with Cobb angle as the outcome and the four covariates, and we reported the percentage attributed to each covariate and to the residual component. The analyses used complete cases and were restricted to the last follow-up outcome.

### Statistical analysis

Continuous variables are presented as mean (SD). Between-group comparisons used Welch’s *t*-test. Ordered trends were evaluated using the Jonckheere–Terpstra test. Multivariable linear regression models were adjusted for age and BMI, with the Cobb angle at the last follow-up as the primary outcome. All tests were two-sided, and a *P*-value less than 0.05 was considered statistically significant. Age at menarche and family history were treated as distinct primary hypotheses in relation to curve severity, and significance for these primary hypotheses was determined after Bonferroni correction for the number of codings of the predictor tested, corresponding to a threshold of *P* less than 0.025. The genetic correlation, Mendelian randomization, and variant-level analyses were methodologically independent analyses addressing different questions, and the null hypothesis was evaluated separately for each rather than pooled into a single correction across all analyses. Analyses of AIS susceptibility were included as control comparisons.

All analyses were performed in R version 4.2.1 (The R Foundation for Statistical Computing, Vienna, Austria). Genetic correlations were estimated using bivariate LD score regression (LDSC). Two-sample MR analyses were conducted using the TwoSampleMR package. Horizontal pleiotropy and robustness were assessed using MR-Egger, the weighted median method, leave-one-out analyses, and MR-PRESSO. Variance partitioning of the last follow-up Cobb angle was performed using the variancePartition package to estimate the proportion of variance explained by family history, AAM, BMI, and age using complete cases. For the relationship between AIS susceptibility and curve severity, harmonized SNP-specific effect estimates were compared using unweighted linear regression, and sign concordance was assessed with a binomial test.

## Results

### Dose-dependent associations of family history and Cobb angle in AIS

We first analyzed the association between family history and Cobb angle. Among the 5,880 subjects with available information on family history of AIS, 1,231 (21.0%) reported a positive family history. Of these, 158 patients (12.8% of those with a positive family history; 2.7% of the overall cohort) had multiple affected relatives.

**Figure 1:**
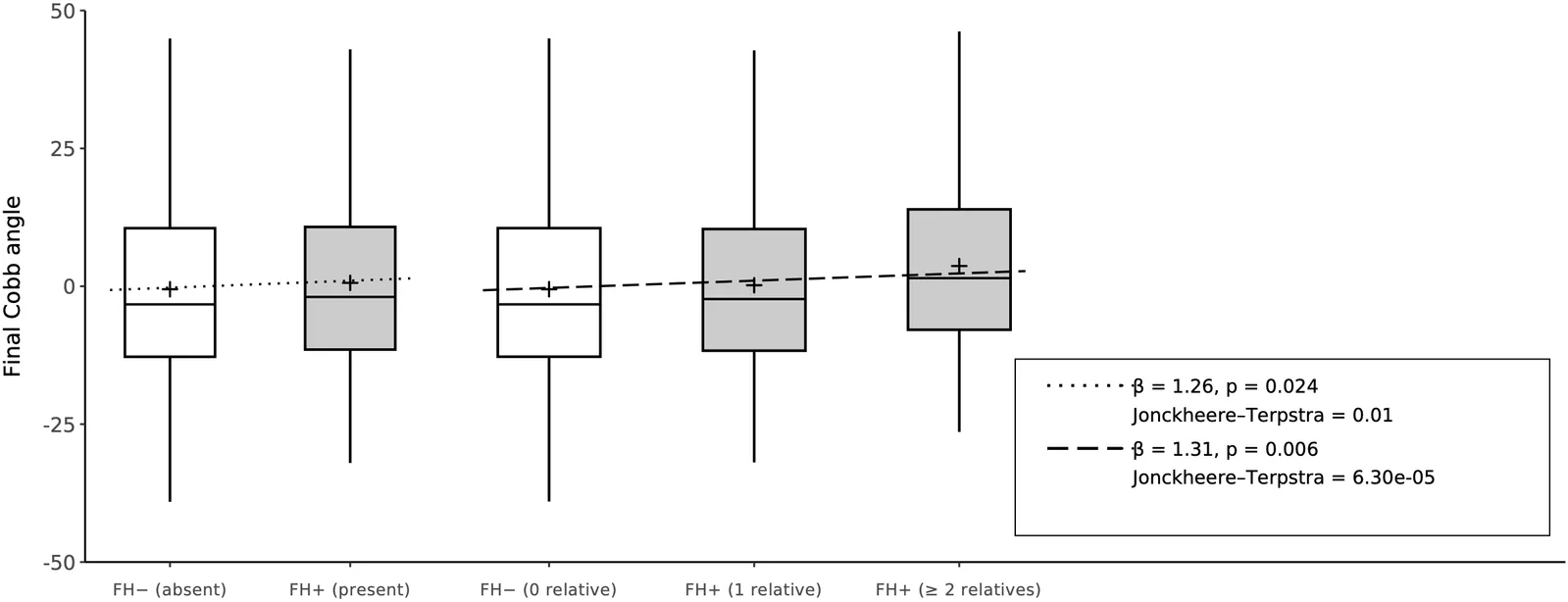
FH and Cobb angle at the final follow-up. Residuals of the Cobb angle at the final follow-up after regression on age and BMI, displayed by FH status on the left (FH absent; *n* = 3,929 vs. FH present; *n* = 997) and by ordered categories of the number of affected close relatives on the right (zero affected close relatives; *n* = 3,929, one affected close relative; *n* = 874, and ≥2 affected close relatives; *n* = 123) (total *n* = 4,926). Group differences were assessed with linear models and monotonic trends using the Jonckheere–Terpstra test. FH, family history; BMI, body mass index.

We found a significant association between a positive family history of AIS and a greater Cobb angle (*β* = 1.26; *P* = 0.024; **Figure 1**). When we divided the subjects with a positive family history into two groups based on number of family members with AIS (single or multiple family members with AIS) and analyzed a dose-dependent association, we observed a significant association (*β* = 1.31 per category; *P* = 0.006; Jonckheere–Terpstra test, *P* = 6.30 × 10^−5^, **Figure 1**). These results indicate that a positive family history was dose-dependently associated with severe Cobb angle.

### Positive associations of AAM with Cobb angle in AIS

**Figure 2:**
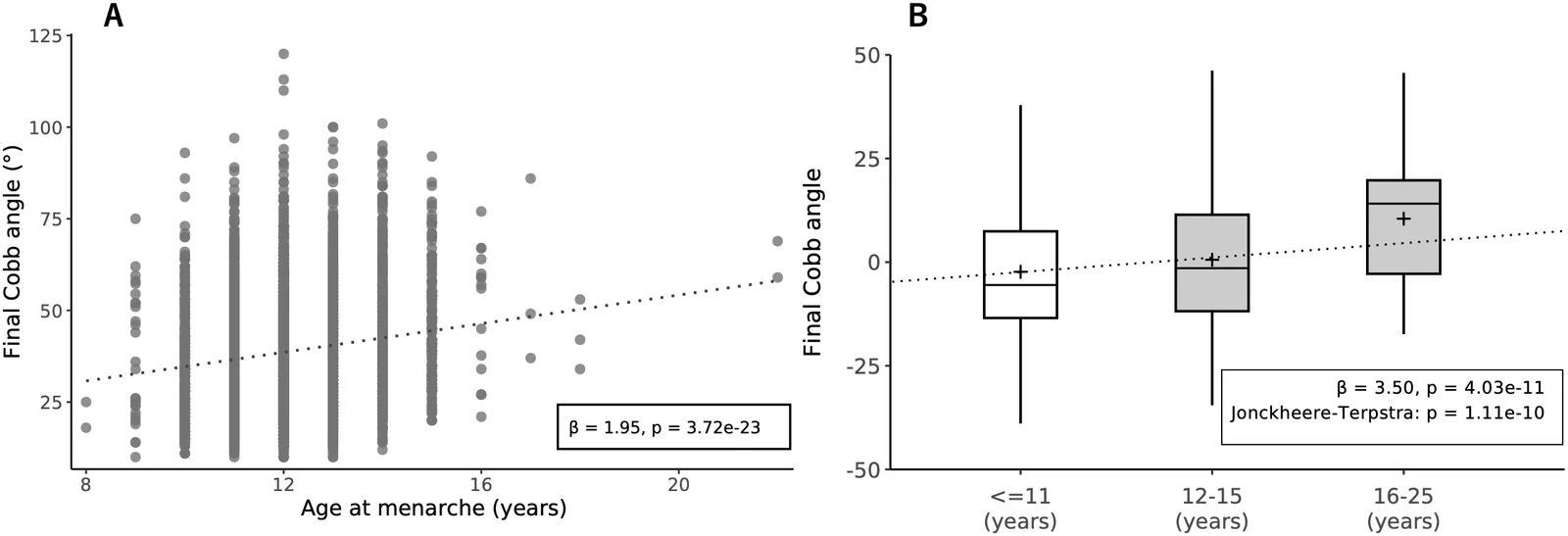
Association of final follow-up Cobb angle with AAM and ordered AAM groups in AIS. **(A)** Cobb angle at the last follow-up versus AAM. The dashed line shows the adjusted fit controlling for age and BMI at the last follow-up. **(B)** Cobb angle at the last follow-up across AAM categories. The values plotted are residuals from a linear regression of the last follow-up Cobb angle on age and BMI at the last follow-up. Boxplots summarize each AAM group: early (≤11 years; *n* = 1,304), control (12–15 years; *n* = 2,792), and late (≥16 years; *n* = 23) (total *n* = 4,119). Group differences were assessed with linear models and monotonic trends using the Jonckheere–Terpstra test. AAM, age at menarche; BMI, body mass index.

Next, we analyzed whether AAM was associated with Cobb angle. We found that later AAM was significantly associated with the Cobb angle (*β* = 1.95; *P* = 3.72 × 10^−23^, **Figure 2A**), indicating that later AAM is linked to AIS curve severity. This result was in line with the previous studies. When we divided the subjects into three groups based on AAM, as expected, the AAM categories showed a strong dose-dependent association with the Cobb angle (*β* = 3.50 per category; *P* = 4.03 × 10^−11^; Jonckheere–Terpstra *P* = 1.11 × 10^−10^; **Figure 2B**). This corresponds to an average of 7° lower Cobb angle in the early group than in the late group.

We also examined the extreme ends of AAM using 1-year AAM bins with at least 10 patients per bin. Consistent with the overall trend, a later AAM was associated with a larger Cobb angle. After adjustment for age and BMI, the latest bin at 16 years showed a greater Cobb angle than the earliest bin at 9 years, with an adjusted difference of 11.78°.

### Positive genetic correlations of AAM with Cobb angle in contrast to AIS susceptibility

Since AAM is also a highly heritable trait, we next investigated the shared genetic architecture between AAM and Cobb angle. Genome-wide genetic correlation analyses using LDSC with an EAS reference showed a significant positive genetic correlation between AAM and AIS curve severity (rg = 0.25; *P* = 0.04, **Table 2**). In contrast, no significant genetic correlation was observed between AAM and AIS susceptibility (rg = 0.04; *P* = 0.33, **Table 2**), suggesting that AAM-related genetic factors are closely shared with AIS curve severity, but not with AIS susceptibility.

**Table 2:** Genetic correlations of AAM with AIS curve severity and AIS susceptibility.

Phenotype 1	Phenotype 2	rg (SE)	95% CI	*Z* score	*P*-value
AAM	AIS curve severity	0.25 (0.12)	0.02–0.49	1.98	0.04*
AAM	AIS susceptibility	0.04 (0.04)	–0.04–0.12	0.96	0.33

Genetic correlations (rg) were estimated using bivariate LD score regression with an East Asian LD reference.

rg, genetic correlation; SE, standard error; CI, confidence interval; AAM, age at menarche; AIS, adolescent idiopathic scoliosis.

*P < 0.05.

### Genetic analyses are consistent with an effect of AAM and a positive variant-level relationship of AIS susceptibility with curve severity

**Figure 3:**
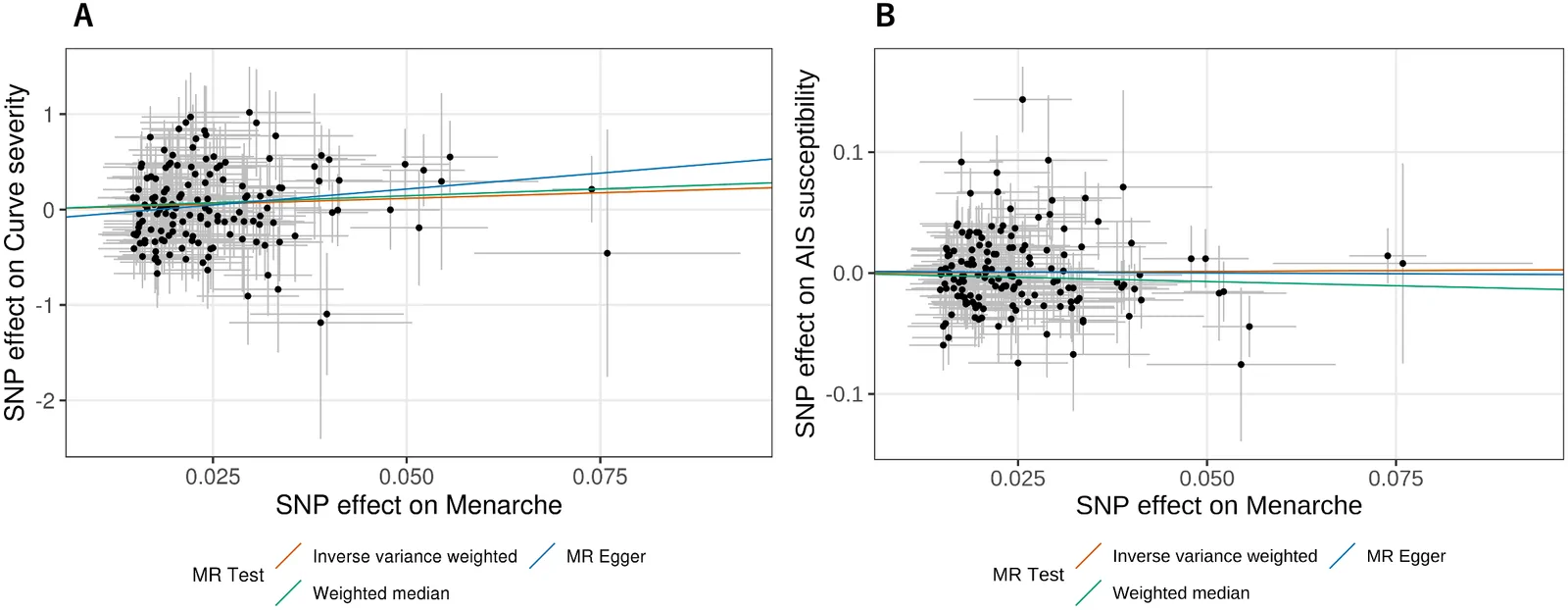
MR analyses of AAM for curve severity and AIS susceptibility. **(A)** Regression plot of MR analyses. Dots represent AAM-associated SNPs plotted with their effect estimates on AAM (x-axis, years) and on curve severity (y-axis, degrees), with 95% confidence intervals. Regression lines are shown for IVW (red), MR-Egger (blue), and weighted median (green). **(B)** Regression plot of MR analyses. Dots represent AAM-associated SNPs with their effect estimates on AAM on the x-axis in years and on AIS susceptibility on the y-axis in log odds with 95% confidence intervals. Regression lines are shown for IVW (red), MR-Egger (blue), and the weighted median (green). MR, Mendelian randomization; AAM, age at menarche; SNP, single-nucleotide polymorphism; IVW, inverse variance weighted.

The MR analyses used 149 SNPs from the meta-GWAS of AAM as instruments (“Materials and methods”). Using the IVW method, we found suggestive evidence of a positive effect of genetically later AAM on AIS curve severity (estimate = 2.35, *P* = 0.04, **Figure 3**, **Table 3**). The IVW association was nominally significant. In sensitivity analyses, MR-Egger and the weighted median produced estimates in the same direction as IVW, although the weighted median did not reach statistical significance (MR-Egger estimate = 6.67, *P* = 0.04; weighted median estimate = 2.89, *P* = 0.13, **Table 3**). Because the IVW association was only nominally significant and not all sensitivity analyses reached significance, we interpret the overall evidence as suggestive rather than conclusive, with the concordant directions indicating that alleles predisposing to later AAM tend to accompany greater curve severity. Funnel plots, single-SNP forest plots, and leave-one-out analyses (**Supplementary Figures S1A–C**) showed a roughly symmetric dispersion of instrument-specific estimates around the IVW slope and no single variant disproportionately influencing the result, supporting the robustness of these findings. For the curve severity analysis, the diagnostics were consistent with no directional pleiotropy and did not indicate outliers (Egger intercept = -0.11, *P* = 0.15, MR-PRESSO global *P* = 0.52; **Supplementary Table S1**).

**Table 3:** Results of the MR analyses for AAM on curve severity.

Outcome	Outcome cohort	Instrument form of AAM	Number of SNPs	Method	MR		
		Cohort			Estimate	SE	*P*-value
Curve severity	Japanese	Meta	149	IVW	2.35	1.18	0.04*
				Weighted median	2.89	1.91	0.13
				MR-Egger	6.67	3.26	0.04*

Effects are interpreted per unit increase in genetically predicted AAM (years per effect allele). Effects were obtained using inverse variance weighted (IVW), weighted median, and MR-Egger methods implemented in the TwoSampleMR package after restricting instruments to variants with *F* ≥10.

*P < 0.05.

AIS, adolescent idiopathic scoliosis; AAM, age at menarche; MR, mendelian randomization; SE, standard error; IVW, inverse variance weighted.

To genetically support the relationship between AIS susceptibility and AIS curve severity, we further examined the correspondence of harmonized SNP-specific effect estimates between the two traits. Across variants, SNPs associated with increased AIS susceptibility tended to show positive effects on curve severity, and linear regression demonstrated a significant positive relationship between SNP effects on AIS susceptibility and those on curve severity (*β* = 2.30, *P* = 0.002; **Supplementary Figure S2**). This positive relationship persisted after accounting for SNP-specific uncertainty. Both inverse variance weighted regression (*β* = 1.53, *P* = 0.010) and robust regression (*β* = 1.77, *P* = 0.003) yielded significant positive slopes (**Supplementary Table S2**). Consistently, 12 of 17 SNPs (70.6%) showed concordant effect directions across the two traits, although this directional concordance did not reach statistical significance in a binomial test (*P* = 0.07). These findings support a positive genetic relationship, whereby alleles increasing susceptibility to AIS also tended to increase curve severity.

In line with genetic correlation analyses, the MR analysis of the causal effect of AAM on AIS susceptibility did not show statistically significant associations across methods (IVW estimate = 0.03, *P* = 0.77; MR-Egger estimate = -0.03, *P* = 0.91; weighted median estimate = -0.13, *P* = 0.25; **Figure 3**; **Supplementary Tables S3**, **S4**, **Supplementary Figures S3A–C**).

For the AIS susceptibility analysis, the MR-PRESSO global test was significant (*P* < 0.001) and identified a single outlier variant, and the distortion test was also significant (*P* = 0.017), indicating that this variant influenced the point estimate. The estimate remained null after outlier correction (outlier-corrected *P* = 0.98), so the absence of a detectable effect of AAM on AIS susceptibility did not depend on this variant.

Taken together, these results suggest that genetic components shared between AAM and AIS curve severity may be distinct from those shared between AIS susceptibility and AIS curve severity.

### Variance explained by clinical factors in the last follow-up Cobb angle

Next, to quantify the contributions of family history and AAM to AIS curve severity, we conducted variance partitioning analyses (“Materials and methods”). AAM explained 2.7% of the variance in the Cobb angle, followed by age (1.9%), family history (0.2%), and BMI (0.1%) (**Supplementary Figure S4**). Thus, AAM and family history together explained approximately 3% of the variance in curve severity, and most of the variance remained unexplained by these clinical factors.

As a secondary analysis, we evaluated associations between family history, AAM, and Cobb angle at the initial visit to the hospitals. We did not observe a clear association between a positive family history of AIS and the initial Cobb angle (*β* = 0.23; *P* = 0.77; **Supplementary Figure S5**). When we divided the subjects with a positive family history into groups based on the number of affected family members, the graded association with initial Cobb angle remained weak (*β* = 0.35 per category; *P* = 0.59; Jonckheere–Terpstra *P* = 0.54; **Supplementary Figure S5**).

In contrast, later AAM was significantly associated with a greater initial Cobb angle with a weaker effect size (*β* = 1.58; *P* = 2.92 × 10^-8^; **Supplementary Figure S6**) than with a final Cobb angle, and AAM categories showed a dose-dependent association (*β* = 1.75 per category; *P* = 0.02; Jonckheere–Terpstra *P* = 0.006; **Supplementary Figure S6**), consistent with but weaker than the pattern seen for the last follow-up Cobb angle.

In treatment-stratified sensitivity analyses, the associations of family history (**Supplementary Figures S7**, **S8**) and age at menarche (modeled continuously, **Supplementary Figure S9**) with the final Cobb angle were preserved within both conservatively managed and surgically treated patients.

## Discussion

In this study, we examined how quantitatively family history and AAM relate to curve severity in AIS. We first focus on clinical signals observed in our large longitudinal AIS cohort and then extend the interpretation to genetics.

First, family history was associated with curve severity. In our cohort, 21% of patients had a positive family history, which is slightly lower than 26%–51% as reported in previous studies (12, 18). This difference may be attributable to variations in study populations and definitions. Prior reports generally assessed family history in broader idiopathic scoliosis cohorts that included both juvenile and adolescent cases, whereas we restricted our analysis to AIS and conservatively classified ambiguous cases as negative, which may have contributed to the lower prevalence observed in our cohort.

Both the presence and intensity of family history were associated with greater curvature after adjustment for age and BMI, with a clear dose–response pattern across ordered categories. The mean Cobb angle increased by about 1.3° for each one-category increase in the number of affected relatives. Taken together, family history was associated with curve severity, but the magnitude of the association was small and its predictive value at the individual level was limited, with family history explaining only 0.2% of the variance in the Cobb angle. Family history should therefore be regarded as one of several modest correlates rather than a standalone predictor of severity, although, because it is easily obtained, it may still add context within a broader assessment.

AAM and AAM classification showed strong associations with AIS curve severity. When AAM was stratified into three categories, the late AAM group had approximately a 7° larger Cobb angle than the early AAM group. This pattern was reinforced by extreme AAM bin analyses. These findings indicate that late AAM linearly relates to greater curve severity.

Because AIS typically emerges around puberty, some patients may be premenarchal at the hospital visits. Monitoring menarche status during follow-up may therefore aid clinical risk assessment. In addition, because AAM is heritable, asking about a family history of AAM may provide useful context when a patient’s own AAM is not yet known.

We found that these associations were not observed or were observed in a weaker manner for Cobb angle at the initial visits. These can be interpreted by the variable timing of patients with AIS to visit hospitals to see medical doctors, independently from the severity of AIS.

In our variance partitioning analysis, AAM explained the largest share of Cobb angle among the clinical covariates, although the absolute contributions were modest. Importantly, family history is a coarse proxy for genetic predisposition and is unlikely to capture the full polygenic architecture of AIS curve severity. Consistent with this, a GWAS-based analysis reported a measurable SNP heritability for the Cobb angle of 21% (17), suggesting that substantial genetic components underlying AIS curve severity extend beyond what can be captured by family history alone.

A number of clinical studies have linked AIS to AAM and reported that later AAM is associated with greater Cobb angle and faster progression (19–21). In parallel, genetic and epidemiologic work has suggested relationships between AIS susceptibility and several traits, including low BMI (10, 39) and BMD (40, 41). However, few studies have addressed whether genetic predisposition contributes to AIS curve severity rather than AIS onset. This gap highlights the need to clarify the genetic overlap between AIS susceptibility and curve severity and to independently assess the genetic overlap between AAM and AIS curve severity. These clinical signals motivated our focus on severity as the primary endpoint in the genetic analyses.

While we also showed that SNPs associated with AIS susceptibility tended to have concordant effects on curve severity, our genetic findings suggest that later AAM is more strongly linked to AIS curve severity than to AIS onset. This distinction implies that AAM may act as a modifier of severity among individuals who develop AIS rather than a primary determinant of susceptibility to AIS. Based on the available evidence, our findings provide genetic support for a contribution of AAM to AIS curve severity.

Clinically, menarche is a practical marker of pubertal maturation in AIS. As highlighted in longitudinal studies and clinical reviews of puberty-related AIS progression, curve progression is strongly influenced by growth velocity and remaining growth potential, and the most rapid progression is generally expected to occur before or around menarche (20, 21). After menarche, growth rate typically slows and residual growth narrows, which help explain why curves tend to stabilize over time. Within this framework, later AAM may correspond to a longer period of skeletal immaturity before curves stabilize, offering a clinical context for our observation that later AAM is associated with greater final curvature.

From a biological perspective, AAM reflects that estrogen regulated maturation (22–24), and prior work has linked estrogen receptor signaling to AIS progression and severity. A systematic review and meta-analyses of estrogen receptor gene variants reported that polymorphisms in nuclear estrogen receptors are associated with curve progression rather than susceptibility (25). GPER1, which is a membrane G protein coupled estrogen receptor, shows genomic polymorphisms associated with curve severity, but not with disease susceptibility, in AIS cohorts (26). ESR1, which is the nuclear estrogen receptor alpha, exhibits asymmetric signaling in paraspinal muscle progenitors in AIS, and the pharmacologic rescue of this asymmetry reduced progression in a mouse model (27). In human paravertebral muscle, the expression of ESR1 and ESR2 shows side-to-side asymmetry related to the AIS phenotype (28). Taken together, these findings link estrogen receptor signaling to AIS progression and severity rather than susceptibility, providing a biologically plausible framework for our genetic evidence that later AAM contributes to greater curve severity.

There are several limitations in our study. Our analyses were conducted in East Asian cohorts, with the clinical cohort and the Cobb angle-based outcome GWAS derived primarily from Japanese patients. Generalizability to other populations is therefore uncertain, and replication in non-East Asian cohorts is needed. Although we restricted AAM instruments to variants present in East Asian data with concordant directions, differences in LD and allele frequencies across ancestries may affect instrument performance and transportability. The outcome GWAS for AIS curve severity had a smaller sample size than large susceptibility GWAS, which may limit power and precision. For the curve severity analysis, MR-Egger and MR-PRESSO did not indicate substantial pleiotropy or outliers, whereas the susceptibility analysis showed significant global heterogeneity driven by a single outlier variant that did not alter the null result. Residual pleiotropy or other violations of MR assumptions cannot be completely excluded. In the clinical analyses, the late AAM group (≥16 years) was small (*n* = 23), so category-specific estimates for this group should be interpreted with caution. The continuous variable analysis, which does not rely on this grouping, gave consistent results. Our models were adjusted for age and BMI. We did not include baseline Cobb angle or treatment as covariates because both lie downstream of curve severity and conditioning on them could introduce over-adjustment, and Risser grade was not fully recorded. Additionally adjusting for follow-up duration did not substantially change the associations of family history or age at menarche with the final Cobb angle, so the primary models were adjusted for age and BMI only. Some residual confoundings may therefore remain. Finally, measurement differences in AAM and in radiographic endpoints, as well as treatment heterogeneity during follow-up, may have introduced additional noise that attenuated the estimated associations.

## Conclusion

In patients with AIS, we found that both a greater family history burden and a later AAM were associated with curve severity. Genetic correlation analyses indicated a positive genetic correlation between AAM and AIS curve severity, with no significant correlation for AIS susceptibility. Furthermore, MR analyses provided suggestive evidence consistent with an effect of genetically later AAM on curve severity, while our MR estimates of AAM on AIS susceptibility were not significant. These genetic findings provide support, at the genetic level, for long-standing clinical observations that later AAM is linked to greater curvature. We also found that SNPs associated with AIS susceptibility tended to show positive effect sizes on AIS curve severity, indicating the possibility that different genetic components underlying AAM and AIS susceptibility were shared by AIS curve severity.

To the best of our knowledge, this is the first report to demonstrate these relationships at the genetic level. Taken together, our clinical and genetic findings highlight family history and AAM as important correlates of AIS curve severity. Recognizing these links may improve risk awareness and help refine risk stratification and individualized follow-up planning.

## Data Availability

The datasets generated and/or analyzed during the current study are available from the corresponding author upon reasonable request, subject to ethical and institutional restrictions.
