# Supplementary material for: Clinical and genetic evidence from East Asian cohorts linking family history burden and menarche timing to curve severity in adolescent idiopathic scoliosis
Source: Front Endocrinol (Lausanne). 2026 Aug 7;17:1876849. doi: 10.3389/fendo.2026.1876849 (PMC13493220; doi:10.3389/fendo.2026.1876849)
Supplement: Supplementary file 1 [file DataSheet1.docx]

**Supplementary Material**

**Supplementary Methods**

**1. Genetic correlation between AAM, Curve severity, and AIS susceptibility**
1.1 GWAS data sources	3
1.2 Preprocessing of summary statistics	3
1.3 LD Score regression analyses	4

**2. Mendelian randomization and variant-level genetic analyses of AAM, Curve severity, and AIS susceptibility**
2.1 GWAS data sources	4
2.2 Derivation of AAM exposure instruments	5
　2.2.1 AAM-derived instruments (AAM as exposure)	5
　2.2.2 AIS-susceptibility–derived variants for comparison with Curve severity	6
2.3 Outcome GWAS formatting	6
2.4 Harmonization and instrument strength filtering	7

2.5 Primary analyses	8

2.5.1 Mendelian randomization analyses for AAM	8
2.5.2 Variant-level comparison between AIS susceptibility and Curve severity	8

2.6 Sensitivity and diagnostic analyses	9

**Supplementary Figures**

Figure. S1. Sensitivity analyses for Mendelian randomization of AAM on curve severity..11

Figure. S2. Harmonized scatter plot of SNP effects for AIS susceptibility and Curve severity	12

Figure. S3. Sensitivity analyses for Mendelian randomization of AAM on AIS susceptibility	13
Figure. S4. Variance explained by clinical factors in the last-follow-up Cobb angle	14

Figure. S5. Initial visit associations with the Cobb angle	15

Figure. S6. Association of initial Cobb angle with AAM and ordered AAM groups in AIS
16

Figure. S7. Family history and final Cobb angle in conservatively managed patients	17

Figure. S8. Family history and final Cobb angle in surgically treated patients	18

Figure. S9. Age at menarche and final Cobb angle, stratified by treatment	19

**Supplemental Tables**

Table S1. MR-Egger intercepts and MR-PRESSO diagnostics for AAM on Curve severity	20
Table S2. Robustness of the variant-level association between AIS susceptibility and Curve severity (17 SNPs)	21
Table S3. Results of the Mendelian randomization analysis of AAM on AIS susceptibility	22

Table S4. MR-Egger intercepts and MR-PRESSO diagnostics for AAM on AIS susceptibility	23

**Supplementary Methods**

**1. Genetic correlation between AAM, Curve severity, and AIS susceptibility**

**1.1 GWAS data sources**
Genetic correlations were estimated using bivariate Linkage Disequilibrium Score regression (LDSC) implemented in the LDSC software (version 1.0.1) ^(1,2)^. We analyzed summary statistics for age at menarche (AAM), Curve severity, and Adolescent Idiopathic Scoliosis (AIS) susceptibility. For AAM, we used a large Japanese genome-wide association study (GWAS) of reproductive ageing that included up to 67,029 women of Japanese ancestry ^(3)^. For Curve severity, we used GWAS summary statistics from an East Asian AIS cohort of 4,465 patients in which the maximum Cobb angle was analyzed as a quantitative severity phenotype ^(4)^. For AIS susceptibility, we used a Japanese case–control GWAS comprising 5,327 AIS cases and 73,884 controls ^(5)^. All three GWASs were conducted in East Asian populations, and the reported effect allele was treated as the effect allele for each trait.

**1.2 Preprocessing of summary statistics**
Before LDSC, summary statistics for each trait were standardized using the munge_sumstats.py utility distributed with LDSC (available at https://github.com/bulik/ldsc). This preprocessing mapped the SNP identifier, effect and non-effect alleles, effect size, and P value into the LDSC input format, restricted variants to HapMap3 SNPs, aligned alleles to a common reference strand, and removed strand ambiguous palindromic variants with intermediate allele frequencies. Analyses were restricted to autosomal SNPs, and the major histocompatibility complex region on chromosome 6 (approximately 25–34 Mb) was excluded because of its complex LD structure.

**1.3 LDSC analyses**
Bivariate LDSC was then performed with the --rg option to estimate genetic correlations (rg) between AAM and Curve severity and between AAM and AIS susceptibility. East Asian LD scores derived from 1000 Genomes East Asian samples were used both as the LD reference and as regression weights to match the ancestry of the GWAS data. For each trait pair, LDSC produced an estimate of rg with its standard error, a z statistic, and a two-sided P value, together with LDSC intercepts and SNP heritability estimates for each trait. Intercepts were examined to assess potential confounding from population stratification or sample overlap.

**2. Mendelian randomization and variant-level genetic analyses of AAM, Curve severity, and AIS susceptibility**

We conducted genetic analyses to evaluate the relationships among AAM, AIS susceptibility, and Curve severity. Specifically, we performed two-sample Mendelian randomization (MR) analyses for AAM in relation to Curve severity and AIS susceptibility, and a harmonized variant-level comparison between AIS susceptibility and Curve severity.

**2.1 GWAS data sources**

We used GWAS summary statistics for three traits. AAM was obtained from a large multi-ancestry meta-analysis including 799,845 women, of whom 166,890 were of East Asian ancestry from cohorts such as the China Kadoorie Biobank, Biobank Japan, and the Korean Genome and Epidemiology Study ^(6)^. For MR, we used effect estimates and allele frequencies from the East Asian subset of this meta-analysis. For Curve severity in AIS, we used GWAS summary statistics from an East Asian AIS cohort of 4,465 patients in which the maximum Cobb angle was analyzed as a quantitative severity phenotype ^(4)^. For AIS susceptibility, we used a Japanese case–control GWAS comprising 5,327 AIS cases and 73,884 controls ^(5)^.

**2.2 Derivation of AAM exposure instruments**

We derived genetic instruments separately for AAM and for AIS susceptibility, corresponding to analyses in which each trait was treated as the exposure.

**2.2.1 AAM-derived instruments (AAM as exposure)**

We treated AAM as the exposure and considered Curve severity and AIS susceptibility as outcomes in separate two-sample MR analyses. Candidate genetic instruments for AAM were derived from the meta-analysis ^(6)^, which reported 1,080 genome-wide significant signals (P < 5×10⁻⁸). For each of these 1,080 SNPs, we assembled the rsID, chromosome, position, alleles, and effect estimates from both the multi-ancestry meta-analysis and the East Asian subset, including the East Asian standard errors and P values. We then restricted the analysis to SNPs with finite, non-zero effect estimates in both datasets and concordant directions of effect. In other words, the estimated effects on AAM had the same sign in the meta-analysis and in the East Asian subset. This procedure removed SNPs with imprecise or discordant effects between the multi-ancestry meta-analysis and the East Asian subset, yielding 799 East Asian–concordant AAM-associated variants. For MR, we used the effect size and standard error from the East Asian subset as the SNP–exposure association, together with the East Asian effect allele frequency. In all analyses, these exposure effect sizes (βₓ) represent the per-allele change in AAM measured in years, consistent with the definition used in the main manuscript.

**2.2.2 AIS-susceptibility–derived variants for comparison with Curve severity**

To evaluate the relationship between AIS susceptibility and Curve severity at the variant level, we started from the Japanese AIS case–control GWAS ^(5)^ and selected 20 genome-wide significant variants (P < 5×10⁻⁸). For each variant, we extracted the rsID, effect allele, non-effect allele, effect allele frequency, effect estimate, standard error, and P value from the AIS susceptibility GWAS. These variants were then matched to the Curve severity GWAS summary statistics so that harmonized SNP-specific effect estimates could be compared between the two traits.

**2.3 Outcome GWAS formatting**
For Curve severity, we used summary statistics with per-allele effect estimates expressed as changes in Cobb angle (degrees), along with standard errors and P values.

For AIS susceptibility, the original GWAS reported odds ratios; we converted these to log-odds effects (β = log(OR)) and derived standard errors from the reported two-sided P values under a normal approximation. Specifically, for each variant we converted the P value to a z-statistic (assuming a standard normal distribution) and calculated the standard error as SE = |β|/|z|. Variants with non-finite β, SE, or P were excluded.

The final outcome datasets included, for each SNP, the effect and non-effect alleles, β (degrees for Curve severity; log odds for AIS susceptibility), SE, and P value.

**2.4 Harmonization and instrument strength filtering**
All MR analyses were performed in R using the TwoSampleMR package ^(7)^. For AAM-based analyses, we began from the common list of 799 East Asian–concordant AAM instruments and restricted each outcome dataset (Curve severity or AIS susceptibility) to SNPs present in the exposure dataset. For AIS-susceptibility–based analyses, we began from the set of genome-wide significant AIS susceptibility variants and restricted the Curve severity summary statistics to SNPs available in this exposure dataset.

Exposure and outcome datasets were harmonized in R using the TwoSampleMR harmonise_data function. This procedure aligns exposure and outcome effect alleles to a common reference, flips the sign of effect estimates where necessary, and removes ambiguous palindromic variants with intermediate allele frequencies to minimize strand alignment errors. We then applied the default TwoSampleMR instrument filter to retain only variants deemed suitable for MR after harmonization. Instrument strength was quantified using SNP-level F statistics defined as F = (βₓ / SE(βₓ))², where βₓ and SE(βₓ) are the effect estimate and its standard error for the association between the SNP and AAM in the East Asian subset. For each outcome, we retained only variants with F ≥ 10 to reduce weak-instrument bias. After harmonization and F-statistic restriction, we obtained 149 independent AAM instruments for Curve severity and 150 instruments for AIS susceptibility. For the AIS susceptibility–Curve severity analysis, we started from 20 genome-wide significant variants from the AIS susceptibility GWAS. Of these, 3 variants were not present in the Curve severity GWAS summary statistics, leaving 17 variants that could be harmonized and included in the variant-level comparison.

**2.5 Primary analyses**

**2.5.1 Mendelian randomization analyses for AAM**

For each outcome we estimated the causal effect of genetically proxied AAM using inverse-variance weighting (IVW) as the primary MR method ^(8)^. SNP-specific causal estimates were obtained as Wald ratios, βᵧ / βₓ, where βᵧ is the SNP–outcome effect and βₓ is the SNP–AAM effect.

When AAM was used as the exposure, effect sizes were interpreted per unit increase in genetically predicted AAM (years per effect allele). In analyses using Curve severity as the outcome, the IVW estimate therefore represents the mean difference in maximum Cobb angle, in degrees, per unit increase in genetically predicted AAM (years per effect allele). In analyses using AIS susceptibility as the outcome, the IVW estimate represents the corresponding change in the log odds of AIS per unit increase in genetically predicted AAM, and these effects are also presented as odds ratios with 95% confidence intervals for interpretability.

**2.5.2 Variant-level comparison between AIS susceptibility and Curve severity**

To evaluate the relationship between AIS susceptibility and Curve severity, we compared harmonized SNP-specific effect estimates between the two traits at the variant level. Specifically, the SNP effect estimates for AIS susceptibility (x-axis; log-odds scale) and the corresponding SNP effect estimates for Curve severity (y-axis; degrees) were plotted for the 17 variants available in both datasets after allele harmonization. We then fitted an unweighted linear regression of SNP effects on Curve severity against SNP effects on AIS susceptibility. The resulting regression slope was used to quantify the overall direction and magnitude of the cross-trait relationship across variants. In this primary analysis, each SNP contributed equally, and no inverse-variance weighting was applied. To examine whether this relationship was sensitive to SNP-specific uncertainty or to individual variants, we additionally fitted an inverse-variance weighted regression, in which each variant was weighted by the inverse of the squared standard error of its Curve severity effect, and a robust regression using an M-estimator, which down-weights influential points. We considered the relationship robust if the slope remained positive and consistent across these analyses.

**2.6 Sensitivity and diagnostic analyses**

To assess the robustness of the AAM-based MR findings and potential violations of MR assumptions, we performed several sensitivity and diagnostic analyses for both outcomes. In addition to IVW, we fitted MR-Egger regression with an unconstrained intercept ^(9)^ and the weighted median estimator ^(10)^. MR-Egger relaxes the assumption of no directional pleiotropy and allows the average direct effect of the instruments on the outcome to differ from zero. We report both the MR-Egger slope and the intercept, where a non-zero intercept was interpreted as evidence of directional horizontal pleiotropy.

We next conducted single-SNP and leave-one-out analyses. Single-SNP MR provided a causal estimate for each instrument individually and was summarized in forest plots. Leave-one-out analyses for the IVW estimator were performed by iteratively excluding each SNP and refitting the IVW model. Funnel plots, forest plots, and leave-one-out plots were inspected to determine whether the overall causal estimate was driven by a small number of variants or by asymmetry compatible with pleiotropy.

Finally, we applied MR-PRESSO (Mendelian Randomization Pleiotropy Residual Sum and Outlier) to detect and correct for horizontal pleiotropic outliers ^(11)^. For each outcome, the global test was used to evaluate the presence of outlier variants. When outliers were detected, we additionally performed the MR-PRESSO distortion test to assess whether these variants significantly altered the estimate, and we recalculated the IVW estimate after excluding them, reporting the outlier-corrected estimate together with the original estimate.

As an additional directional analysis for the AIS susceptibility–Curve severity comparison, we assessed sign concordance between SNP effects on AIS susceptibility and Curve severity. Variants were classified as concordant when the SNP effects had the same direction for both traits, and the observed proportion of concordant variants was compared with the null expectation of 0.5 using a binomial test.

**Supplementary Figure S1. Sensitivity analyses for Mendelian randomization of AAM on Curve severity.**

**A–C** **Sensitivity analyses for the causal effect of AAM on Curve severity.
A.** Funnel plot showing single variant causal estimates against their precision. The symmetry of the plot and the overlap of regression lines for IVW, MR-Egger, and weighted median methods indicate the absence of directional pleiotropy.
**B.** Forest plot presenting single variant causal estimates with 95% confidence intervals for IVW, MR-Egger, and weighted median analyses. The consistent direction of effects supports the robustness of the IVW result.
**C.** Leave-one-out analysis showing that exclusion of any single variant did not materially alter the overall IVW estimate, confirming the stability of the causal association between genetically later AAM and greater Curve severity.

**Supplementary Figure S2. Harmonized scatter plot of SNP effects for AIS susceptibility and Curve severity.**

Shown are harmonized SNP-specific effect estimates for AIS susceptibility (x-axis) and Curve severity (y-axis). Each point denotes one SNP included after harmonization. The solid line represents the unweighted linear regression of SNP effects on Curve severity versus SNP effects on AIS susceptibility. The positive regression slope (β = 2.30, P = 0.002) supports a positive association between genetically increased AIS susceptibility and greater Curve severity.

Abbreviations: AIS, adolescent idiopathic scoliosis; SNP, single nucleotide polymorphism.

**Supplementary Figure S3. Sensitivity analyses for Mendelian randomization of AAM on AIS susceptibility.**

**A–C Sensitivity analyses for the causal effect of AAM on AIS susceptibility.**
**A.** Funnel plot of single variant causal estimates and precision, showing symmetric distribution and regression slopes close to zero across methods.
**B.** Forest plot displaying variant-specific estimates and pooled effects, indicating no consistent evidence of a causal relationship between AAM and AIS susceptibility.
**C.** Leave-one-out analysis showing that the overall estimate remained null when individual variants were excluded, consistent with the outlier-corrected result.

For AAM on AIS susceptibility, the MR-Egger intercept showed no directional pleiotropy (P = 0.82), whereas the MR-PRESSO global test (P < 0.001) and distortion test (P = 0.017) identified a single influential outlier. The estimate remained null after outlier correction (outlier-corrected P = 0.98), indicating that the absence of a detectable effect of AAM on AIS susceptibility did not depend on this variant.

Abbreviations: AAM, age at menarche; AIS, adolescent idiopathic scoliosis; IVW, inverse-variance weighted; MR, Mendelian randomization; SNP, single nucleotide polymorphism; OR, odds ratio.

**Supplementary Figure S4. Variance explained by clinical factors in the last-follow-up Cobb angle**

Variance partitioning with a fixed-effects linear model in R (variance Partition) including AAM, BMI, age, and FH (n = 4121). Bars show the percentage of variance in the last-follow-up Cobb angle attributed to each factor and to residual variation. AAM explained 2.7%, age 1.9%, FH 0.2%, BMI 0.1%, and residuals 95.2%.

Abbreviations: AAM, age at menarche; BMI, body mass index; FH, family history.

**Supplementary Figure S5. Family history and Cobb angle at the initial visit**
Residuals of the Cobb angle at the initial visit after regression on age and BMI, displayed by family history status on the left (family history absent; n = 1726 vs family history present; n = 441) and by ordered categories of the number of affected close relatives on the right (0 affected close relatives; n = 1726, 1 affected close relative; n = 382, and ≥2 affected close relatives; n = 59) (total n = 2167). Group differences were assessed with linear models and monotonic trends with the Jonckheere–Terpstra test. Analyses at the initial visit showed no significant differences across groups.

Abbreviations: FH, family history; BMI, body mass index.

 **Supplementary Figure S6. Association of initial Cobb angle with AAM and ordered AAM groups in AIS**

**A.** Cobb angle at the initial visit versus AAM. The dashed line shows the adjusted fit controlling for age and BMI at the initial visit.

**B.** Cobb angle at the initial visit across AAM categories. Values plotted are residuals from a linear regression of the initial Cobb angle on age and BMI at the initial visit. Boxplots summarize each AAM group: early (≤11 years; n = 562), control (12–15 years; n = 1254), and late (≥16 years; n = 8) (total n = 1824). Group differences were assessed with linear models and monotonic trends with the Jonckheere–Terpstra test.

Abbreviations: AAM, age at menarche; BMI, body mass index.

**Supplementary Figure S7. Family history and final Cobb angle in conservatively managed patients**

Residuals of the final follow-up Cobb angle after regression on age and BMI, displayed by family-history status (family history absent; n = 2453 vs family history present; n = 636) and by the number of affected close relatives (0 affected close relatives; n = 2451, 1 affected close relative; n = 557, and ≥2 affected close relatives; n = 79) (total n = 3087), in conservatively managed patients. Boxes show the residual distribution, the cross marks the mean, and the dotted and dashed lines show the adjusted trends for family-history status and for the number of relatives. β and p are from multivariable linear regression adjusting for age and BMI, and the Jonckheere–Terpstra test assessed the monotonic trend, with values shown in the panel. Family history was significantly associated with a greater final Cobb angle (presence β = 1.36, p = 0.002, and relatives β = 1.36, p = 2.7×10⁻⁴).

Abbreviations: FH, family history; BMI, body mass index.

**Supplementary Figure S8. Family history and final Cobb angle in surgically treated patients**

As in Supplementary Figure S7, but for surgically treated patients (family history absent; n = 1314 vs family history present; n = 324; 0 affected close relatives; n = 1311, 1 affected close relative; n = 283, and ≥2 affected close relatives; n = 41) (total n = 1635). β and p are from multivariable linear regression adjusting for age and BMI, and the Jonckheere–Terpstra test assessed the monotonic trend, with values shown in the panel. Family history remained significantly associated with a greater final Cobb angle (presence β = 1.90, p = 0.008, and relatives β = 1.70, p = 0.005).

Abbreviations: FH, family history; BMI, body mass index.

**Supplementary Figure S9. Age at menarche and final Cobb angle, stratified by treatment**

A. Final Cobb angle versus AAM in conservatively managed patients (n = 2588). The dotted line shows the adjusted fit controlling for age and BMI at the last follow-up.

B. Final Cobb angle versus AAM in surgically treated patients (n = 1375). The dotted line shows the adjusted fit controlling for age and BMI at the last follow-up. β and the p value from multivariable linear regression are shown in each panel.

Abbreviations: AAM, age at menarche; BMI, body mass index.

**Supplementary Table S1. MR-Egger and MR-PRESSO diagnostic statistics for the MR analysis using AAM as the exposure and Curve severity as the outcome.**

| **Outcome** | **Outcome cohort** | **AAM cohort** |  | | | |
| --- | --- | --- | --- | --- | --- | --- |
|  |  |  | **MR-Egger** | | | **MR-PRESSO** |
|  |  |  | **Intercept** | **SE** | **P-value** | **Global test**  **P-value** |
| **Curve severity** | Japanese | Meta | -0.11 | 0.08 | 0.15 | 0.52 |
| Instruments for AAM were derived from the meta-GWAS and, after harmonization, filtered to retain variants with F ≥ 10.  * P value <.05  Abbreviations: AAM, age at menarche; MR, mendelian randomization; SE, standard error. | | | | | | |

**Supplementary Table S2. Robustness of the variant-level association between AIS susceptibility and Curve severity (17 SNPs)**

| **Method** | **β (slope)** | **SE** | **P** |
| --- | --- | --- | --- |
| Unweighted linear regression | 2.30 | 0.61 | 0.002* |
| Inverse-variance weighted | 1.53 | 0.52 | 0.01* |
| Robust regression (M-estimator) | 1.77 | 0.60 | 0.003* |
| Values are the slope of SNP effects on Curve severity regressed on SNP effects on AIS susceptibility for the 17 variants shared between the two datasets. The inverse-variance weighted model weighted each variant by the inverse of the squared standard error of its Curve severity effect, and the robust regression used an M-estimator to down-weight influential points. All three methods gave a positive and significant slope. * P < 0.05  Abbreviations: AIS, adolescent idiopathic scoliosis; SNP, single nucleotide polymorphism; SE, standard error. | | | |

**Supplementary Table S3. The MR estimates for the effect of genetically proxied AAM on AIS susceptibility in the Japanese cohort**

| **Outcome** | **Outcome cohort** | **Instrument form of AAM** | **Number of SNPs** | **Method** | **MR** | | |
| --- | --- | --- | --- | --- | --- | --- | --- |
|  |  | **Cohort** |  |  | **Estimate** | **SE** | **P-value** |
| AIS susceptibility | Japanese | Meta | 150 | IVW | 0.03 | 0.09 | 0.77 |
|  |  |  |  | Weighted median | -0.13 | 0.12 | 0.25 |
|  |  |  |  | MR-Egger | -0.03 | 0.26 | 0.91 |
| Instruments for AAM were derived from the meta-GWAS, harmonized with the AIS susceptibility GWAS, and filtered to retain 150 variants with F ≥10. * *P* value <.05  Abbreviations: AIS, adolescent idiopathic scoliosis; AAM, age at menarche; MR, mendelian randomization; SE, standard error; IVW, inverse variance weighted. | | | | | | | |
|  |  |  |  |  |  |  |  |

**Supplementary Table S4. MR-Egger and MR-PRESSO diagnostic statistics for the MR analysis using AAM as the exposure and AIS susceptibility as the outcome.**

| **Outcome** | **Outcome cohort** | **AAM cohort** | **MR** | | | | | | |
| --- | --- | --- | --- | --- | --- | --- | --- | --- | --- |
|  |  |  | **MR-Egger** | | | **MR-PRESSO** | | | |
|  |  |  | **Intercept** | **SE** | **P-value** | **Global test**  **P-value** | **Distortion test P-value** | **Outlier-corrected estimate** | **Outlier-corrected**  **P-value** |
| AIS susceptibility | Japanese | Meta | 0.002 | 0.006 | 0.82 | <0.001* | 0.017* | -0.001 | 0.98 |
| * *P* value <.05  Abbreviations: AIS, adolescent idiopathic scoliosis; AAM, age at menarche; MR, mendelian randomization; SE, standard error. | | | | | | | | | |

**References**

1. Bulik-Sullivan BK, Loh PR, Finucane HK, et al. LD Score regression distinguishes confounding from polygenicity in genome-wide association studies. *Nat Genet*. 2015;47(3):291-295. doi:10.1038/ng.3211.
2. Bulik-Sullivan B, Finucane HK, Anttila V, et al. An atlas of genetic correlations across human diseases and traits. *Nat Genet*. 2015;47(11):1236-1241. doi:10.1038/ng.3406.
3. Horikoshi M, Day FR, Akiyama M, et al. Elucidating the genetic architecture of reproductive ageing in the Japanese population. *Nat Commun*. 2018;9(1):1977. doi:10.1038/s41467-018-04398-z.
4. Otomo N, Lu HF, Koido M, et al. Polygenic Risk Score of Adolescent Idiopathic Scoliosis for Potential Clinical Use. *J Bone Miner Res*. 2021;36(8):1481-1491. doi:10.1002/jbmr.4324.
5. Kou I, Otomo N, Takeda K, et al. Genome-wide association study identifies 14 previously unreported susceptibility loci for adolescent idiopathic scoliosis in Japanese. *Nat Commun*. 2019;10(1):3685. doi:10.1038/s41467-019-11596-w.
6. Kentistou KA, Kaisinger LR, Stankovic S, et al. Understanding the genetic complexity of puberty timing across the allele frequency spectrum. *Nat Genet*. 2024;56(7):1397-1411. doi:10.1038/s41588-024-01798-4.
7. Hemani G, Zheng J, Elsworth B, et al. The MR-Base platform supports systematic causal inference across the human phenome. *Elife*. 2018;7:e34408. doi:10.7554/eLife.34408.
8. Burgess S, Thompson SG. Use of allele scores as instrumental variables for Mendelian randomization. *Int J Epidemiol*. 2013;42(4):1134-1144. doi:10.1093/ije/dyt093.
9. Bowden J, Davey Smith G, Burgess S. Mendelian randomization with invalid instruments: effect estimation and bias detection through Egger regression. *Int J Epidemiol*. 2015;44(2):512-525. doi:10.1093/ije/dyv080.
10. Bowden J, Davey Smith G, Haycock PC, et al. Consistent Estimation in Mendelian Randomization with Some Invalid Instruments Using a Weighted Median Estimator. *Genet Epidemiol*. 2016;40(4):304-314. doi:10.1002/gepi.21965.
11. Verbanck M, Chen CY, Neale B, et al. Detection of widespread horizontal pleiotropy in causal relationships inferred from Mendelian randomization between complex traits and diseases. *Nat Genet*. 2018;50(5):693-698. doi:10.1038/s41588-018-0099-7.
